# Ethanol Extract of the Flower *Chrysanthemum morifolium* Augments Pentobarbital-Induced Sleep Behaviors: Involvement of Cl^−^ Channel Activation

**DOI:** 10.1155/2011/109164

**Published:** 2011-02-10

**Authors:** Jae-Wook Kim, Jin-Yi Han, Jin Tae Hong, Rihua Li, Jae Soon Eun, Ki-Wan Oh

**Affiliations:** ^1^College of Pharmacy, Chungbuk National University, Cheongju 361-763, Republic of Korea; ^2^Research Institute of Veterinary Medicine, Chungbuk National University, Cheongju 361-763, Republic of Korea; ^3^College of Pharmacy, Woosuk University, Samrye 565-701, Republic of Korea

## Abstract

Dried *Chrysanthemum morifolium* flowers have traditionally been used in Korea for the treatment of insomnia. This study was performed to investigate whether the ethanol extract of *Chrysanthemum morifolium* flowers (EFC) enhances pentobarbital-induced sleep behaviors. EFC prolonged sleep time induced by pentobarbital similar to muscimol, a GABA_A_ receptors agonist. EFC also increased sleep rate and sleep time when administrated with pentobarbital at a subhypnotic dosage. Both EFC and pentobarbital increased chloride (Cl^−^) influx in primary cultured cerebellar granule cells. EFC increased glutamic acid decarboxylase (GAD) expression levels, but had no effect on the expression of *α*1-, *β*2-, and *γ*2-subunits of the GABA_A_ receptor in the hippocampus of a mouse brain. This is in contrast to treatment with pentobarbital, which showed decreased *α*1-subunit expression and no change in GAD expression. In conclusion, EFC augments pentobarbital-induced sleep behaviors; these effects may result from Cl^−^ channel activation.

## 1. Introduction

The flower of *Chrysanthemum morifolium *Ramat. (FC) has been used in oriental countries for hundreds of years and is widely consumed as a medicinal herbal tea [1, 2]. FC is reported to have various biological features including antioxidation [3], cardiovascular protection [4], antitumorgenesis [5], and anti-inflammation [6]. Chrysanthemum species have been shown to contain a wide variety of flavonoids, phenols, and phenolic acids [7]. Significant amounts of flavonoids and hydroxycinnamoylquinic acids are considered to be the biologically active components [4, 5, 8], and the health benefits of FC have been shown to be associated with the flavonoids [9]. In Korea, the dried FC in herbal tea has traditionally been used for the treatment of insomnia [3].

Insomnia symptoms are some of the most frequent sleep complaints in the general population, with an estimated prevalence varying from 10 to nearly 60%, depending in part on the use of varying definitions and data-collection methodologies [10]. Individuals reporting disturbed sleep are more likely to report emotional distress and recurrent health problems [11]. This is not surprising since it has been shown that sleep deprivation has a great impact on the everyday life of healthy subjects, affecting alertness, attention, concentration, cognitive abilities, memory, mood, and pain. 

A variety of modulators of GABA-transmission, including neurosteroids, benzodiazepines, barbiturates, and GABA agonists, have been investigated in both *in vitro* and *in vivo* models. It is now well known that their effects are related to their binding to specific GABA_A_-receptor subtypes. Muscimol and other GABA_A_ receptor agonists that potentiate Cl^−^ influx also cause potentiation of Cl^−^ influx when administered with pentobarbital or other agonists [12]. The sedative and anticonvulsant effects of diazepam and zolpidem are primarily mediated by their interaction with the *α*1-subunit [13]. Benzodiazepines and benzodiazepine-like compounds are the most widely used hypnotics; they primarily act to shorten sleep latency and enhance and consolidate sleep. It was postulated that the hypnotic properties of benzodiazepines, that is, their capacity to shorten sleep latency, to reduce waking after sleep onset, and to consolidate sleep are associated with the *α*1-subunit [14, 15]. The various side effects associated with benzodiazepines stimulated the search for alternative compounds for the treatment of insomnia. Therefore, the goal of this study was to evaluate the hypnotic effects of the ethanol extract of the flower of *C. morifolium* (EFC) on pentobarbital-induced sleep behaviors and to investigate its possible mechanisms.

## 2. Materials and Methods

### 2.1. Animals

Male ICR mice (Samtako, Korea) weighing 20–25 g, in groups of 10–12, were used for behavioral experiments. Animals were housed in acrylic cages (45 × 60 × 25 cm) with water and food available *ad libitum* under an artificial 12-h light/dark cycle (lights on at 7:00) and at a constant temperature (22 ± 2°C). Mice were housed in the departmental room for 1 week before testing to ensure adaptation to the new environment. All of the behavioral experiments were performed between 10:00 and 17:00. All of the experiments involving animals were carried out in accordance with the National Institutes of Health Guide for Care and Use of Laboratory Animals (NIH publication No. 85-23, revised 1985), and the Institutional Animal Care and Use Committee of Chungbuk National University approved the protocol.

### 2.2. Cell Culture

Primary cultures of cerebellar neurons enriched in granule cells were prepared from cerebella of 8-day-old Sprague-Dawley rats as previously described [16]. After 8 days in culture, these cells express functional GABA_A_ receptors, with an expression pattern similar to that of the cerebellum during postnatal development, but different from the pattern observed in the adult rat cerebellum [17]. Briefly, cells were plated (1 × 10^5^ cells per well) in 96-well microplates that had been coated with poly-L-lysine (50 *μ*g/mL; Sigma, St. Louis, MO, USA) and were cultured in Dulbecco's modified Eagle's medium nutrient and Ham's F12 mixture media (Life Technologies, Gaithersburg, MD, USA) supplemented with 10% heat-inactivated fetal bovine serum (Life Technologies), glutamine (2 mM), gentamicin (100 *μ*g/mL), antibiotic-antimycotic solution (10 mL/L; Sigma) and Potassium chloride (25 mM); a high concentration of potassium was necessary to induce persistent depolarization, which promotes the survival of granule cells. Cytosine arabinofuranoside (final concentration, 10 *μ*M; Sigma) was added to cultures 18–24 h after plating, to inhibit the proliferation of nonneuronal cells.

### 2.3. Ethanol Extraction of *C. morifolium*

The plant materials were collected during October 2009 at Jiri Mt., Jeonbuk, Korea. The shade-dried *C. morifolium* flower (100 g) was extracted three times with 70% EtOH at 50°C. The extracts were filtered and concentrated using a rotary vacuum evaporator, followed by lyophilization. The yield of *C. morifolium *flower extracts (EFC) was approximately 2.5%.

### 2.4. Pentobarbital-Induced Sleep

Pentobarbital sodium (Hanlim Pharm. Co., Ltd., Korea) was diluted in physiological saline and administered to each mouse intraperitoneally (i.p.) to induce sleep. EFC was suspended in physiological saline and was administered orally (p.o.) to animals. Muscimol (Sigma, USA) was administered as a reference drug 15 min prior to administration of pentobarbital. All experiments were carried out between 13:00 and 17:00. Animals were food-deprived for 24 h prior to the experiment. Thirty minutes after the oral administration of the test samples, pentobarbital was given to animals placed in a box. Animals that stopped moving around the box, whereas animals that failed to fall asleep within 15 min after pentobarbital administration were excluded from the study [18, 19]. Mice that remained immobile for more than 3 min were judged to be asleep. The time that elapsed from receiving pentobarbital until each animal lost its righting reflex when positioned delicately on its back represented the latency to onset of sleep. The animals were observed constantly, and the time of awakening, characterized by righting of the animal, was noted. Sleep time was defined as the time required for the animal to regain spontaneous movements after having been transferred to the second box.

### 2.5. Measurement of Intracellular Cl^−^ Influx

The intracellular Cl^−^ concentration ([Cl^−^]*i*) of cerebellar granule cells was estimated using Cl^−^ sensitive fluorescence probe N-(ethoxycarbonylmethyl)-6-methyoxyquionolinium bromide (MQAE) according to the method of West and Molloy, with a slight modification [20]. The buffer (pH 7.4) contained the following components: 2.4 mM HPO_4_^2−^, 0.6 mM H_2_PO_4_^−^, 10 mM HEPES, 10 mM D-glucose, and 1 mM MgSO_4_. A variety of MQAE-loading conditions were assessed. The cells were incubated overnight in medium containing 10 mM MQAE (Dojindo, Japan). After loading, the cells were washed three times in the appropriate Cl^−^ containing buffer or Cl^−^-free buffer. The buffer was replaced with buffer with or without the compounds or Cl^−^-free buffer. Repetitive fluorescence measurements were initiated immediately using a FLUOstar plate reader (Excitation wavelength: 320 nm, emission wavelength: 460 nm; BMG LabTechnology, Germany). The data is presented as the relative fluorescence *F*_0_/*F*, where *F*_0_ is the fluorescence without Cl^−^ ions and *F* is the fluorescence as a function of time. The *F*_0_/*F* values were directly proportional to [Cl^−^]*i*.

### 2.6. GAD and GABA_A_ Receptors Subunits Expression

Mice were administered EFC or pentobarbital for 3 days and sacrificed. The mice were decapitated, their brains removed and the hippocampus dissected on ice according to the methods described by Glowinski and Iversen [21] and Segal and Kuczenski [22]. Mouse hippocampus was homogenized with lysis buffer. The extracts were centrifuged at 20,000 × g for 20 min. Equal amounts of proteins were separated on a 10% SDS/polyacrylamide gel and transferred to a nitrocellulose membrane (Hyboud ECL, Amersham Pharmacia Biotech Inc., Piscataway, NJ, USA). The blots were blocked for 2 h at room temperature with 5% (w/v) nonfat dried milk in Tris-buffered saline solution (10 mM Tris, pH 8.0 and 150 mM NaCl) containing 0.1% Tween-20. The membrane was incubated with specific rabbit polyclonal antibodies against GABA_A_ receptor subunits (1 : 1000; Abcam Inc.) for 2 h at room temperature. The blot was then incubated with the corresponding antirabbit IgG-conjugated to horseradish peroxidase (Santa Cruz Biotechnology Inc.). The immunoreactive proteins were detected using the ECL western blotting detection system [23].

### 2.7. Statistical Analysis

The results are presented as the mean ± S.E.M. The significance of the effects of the compounds was assessed using analysis of variance (ANOVA). Where there was significant variability, the individual values were compared using Dunnett's test. For the subhypnotic pentobarbital dosage experiment, Chi-square test was used to compare the proportion of sleep onset between the group treated with a subhypnotic dose of pentobarbital alone and each of the groups that received pentobarbital in combination with another drug.

## 3. Results

### 3.1. Effects of EFC on the Onset and Duration of Sleep in Pentobarbital-Treated Mice

**Figure 1:**
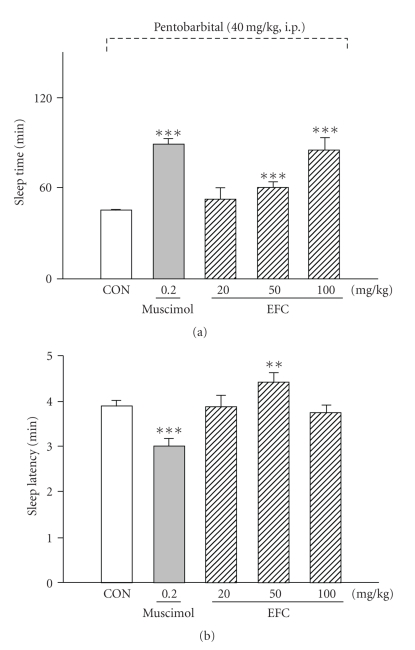
Effects of EFC on onset and duration of sleep in pentobarbital-treated mice. Mice were food-deprived for 24 h before the experiment. Pentobarbital (40 mg/kg, i.p.) was administered to mice following administration of muscimol or EFC. The sleep time (a) and sleep latency (b) were recorded. Each column represents the mean with S.E.M. The significance of the effects of the compounds was assessed using analysis of variance (ANOVA). **P < .01, ***P < .005, compared to control.

The administration of EFC increased sleep time. EFC produced a dose-dependent prolongation of pentobarbital-induced sleep time at dose of 50 mg/kg and 100 mg/kg; however, EFC did not affect the latency of sleep. Pretreatment of mice with muscimol (0.2 mg/kg, i.p.) as a positive control, 15 min before the administration of pentobarbital (40 mg/kg), produced an increase in total sleep time and a decrease in the latency of sleep (Figure 1).

### 3.2. Effects of EFC on Sleep Onset in Mice Treated with a Subhypnotic Dosage of Pentobarbital

Administration of EFC increased the rate of sleep onset and the duration of sleep time induced by a subhypnotic dosage of pentobarbital (28 mg/kg, i.p.). Pretreatment with muscimol also increased the rate of sleep onset and prolonged the duration of sleep time when given in combination with a subhypnotic dosage of pentobarbital (Table 1). EFC showed similar effects to mucimol at 100 mg/kg.

### 3.3. Effects of EFC on Cl^−^ Influx in Primary Cultured Cerebellar Granule Cells

**Figure 2:**
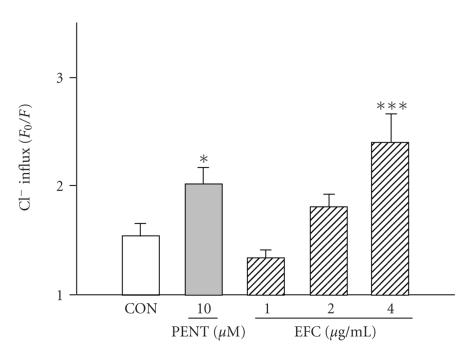
Effects of EFC on chloride influx in primary cultured cerebellar granule cells. After the culture of cerebellar granule cells for 8 days, the cells were incubated with MQAE overnight, and then EFC (1–4 *μ*g/mL) and pentobarbital (PENT, 10 *μ*M) were added 5 min prior to measurement. PENT: pentobarbital. Each column represents the mean with S.E.M. The significance of the effects of the compounds was assessed using analysis of variance (ANOVA). *P < .05, ***P < .005, compared to control.

Intracellular chloride ion influx in primary cultured cerebellar granule cells was measured. The measured data is presented as the relative fluorescence *F*_0_/*F*, where *F*_0_ is the fluorescence without chloride ions and *F* is the fluorescence as a function of each sample. The *F*_0_/*F* values were directly proportional to intracellular chloride ion concentration. Treatment of granule cells with EFC (1–4 *μ*g/mL) produced a significant increase in chloride ion influx. Pentobarbital 10 *μ*M also increased the influx of Cl^−^ in primary cultured cerebellar granule cells (Figure 2).

### 3.4. Effects of EFC on Expression of Glutamic Acid Decarboxylase (GAD) and Subunit of GABA_A_ Receptor

**Figure 3:**
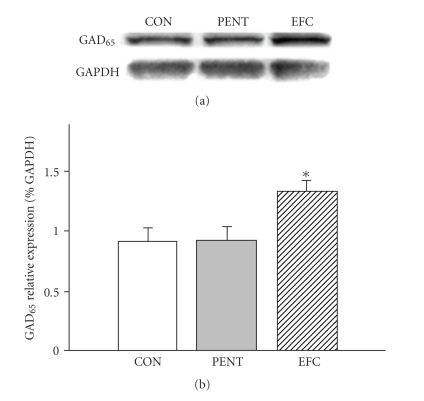
Effects of EFC on glutamic acid decarboxylase (GAD). Immunoblots of lysed mouse hippocampus 3 days following administration of EFC or pentobarbital are shown. GAPDH levels were measured to confirm equal protein loading. PENT: pentobarbital. Each column represents the mean with S.E.M. The significance of the effects of the compounds was assessed using analysis of variance (ANOVA). *P < .05, compared to control.

**Figure 4:**
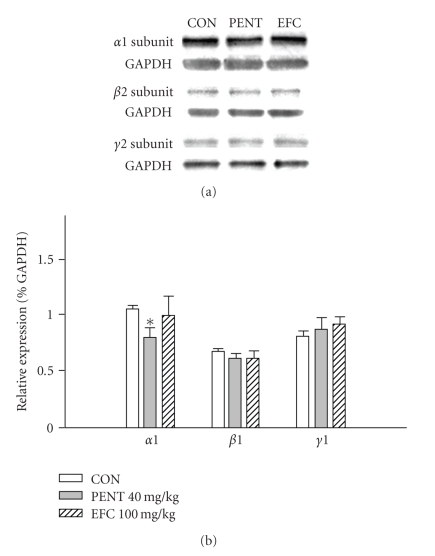
Effects of EFC on GABA_A_ receptor subunits. Immunoblots of lysed mouse hippocampus 3 days following administration of EFC or pentobarbital are shown. GAPDH levels were measured to confirm equal protein loading. PENT: pentobarbital. Each column represents the mean with S.E.M. The significance of the effects of the compounds was assessed using analysis of variance (ANOVA). *P < .05, compared to control.

Mice were administered 100 mg/kg EFC or 40 mg/kg pentobarbital for 3 days, and they were sacrificed to examine the effect of these drugs on the abundance of glutamic acid decarboxylase (GAD) and GABA_A_ receptor subunits in the hippocampus. EFC treatment increased expression of GAD_65_ (Figure 3) but did not influence the amounts of *α*1-, *β*2-, and *γ*2-subunits in the GABA_A_ receptor (Figure 4); however, pentobarbital significantly decreased amounts of the *α*1-subunit, but did not affect the abundance of *β*2- and *γ*2-subunit. Protein concentrations of GAD_65_ following pentobarbital treatment also were not changed.

## 4. Discussion

The results demonstrate that EFC potentiates pentobarbital-induced sleep behaviors in mice. The increase and decrease of pentobarbital-induced sleep time can be a useful tool for examining the stimulatory or inhibitory effects on central nervous system (CNS), especially for investigating drug influences on GABAergic systems [24, 25]. In addition, pentobarbital is well known to potentiate the effects of GABA by acting at its own binding sites on the GABA/benzodiazepine receptor ionophore complex [25]. Many hypnotic, antianxiety and antiepilepsy drugs have been shown to cause prolongation of pentobarbital-induced sleep time [26–28]. We were interested in whether EFC prolongs pentobarbital-induced sleep behaviors and interacts with pentobarbital in the CNS via the GABAergic systems.

We investigated the effects of different doses of EFC and muscimol in rodents with pentobarbital treatment. We found that EFC could potentiate pentobarbital-induced sleep at 100 mg/kg. Additionally, EFC increased the rate of sleep onset and prolonged sleep time at subhypnotic dosages of pentobarbital (28 mg/kg). These results are similar to those of the GABA_A_ receptor agonist muscimol. This indicates that the hypnotic effect of EFC may be due to interaction with GABAergic systems. GABA_A_ receptors possess various binding sites, including binding sites for GABA, benzodiazepine, and barbiturates. GABA_A_ receptors form heteromeric GABA-gated Cl^−^ channels, which are assembled from a large family of subunit genes. GABA_A_ receptor channels open after binding GABA to give a net inward flux of negative Cl^−^ ions (outward current), hyperpolarizing the membrane and reducing neuronal firing [29]. Muscimol and other GABA_A_ receptor agonists that potentiate Cl^−^ influx also cause potentiation of Cl^−^ influx when administered with pentobarbital or other agonists [12]. EFC produced a significant increase in Cl^−^ influx; this increase was similar to that of pentobarbital. This suggests that EFC may act to induce Cl^−^ channel opening of GABA_A_ receptors. 

Researchers have demonstrated that the pharmacological profile and different drug-induced behaviors of GABA_A_ receptors depend upon its subunit composition [30]. Glutamic acid decarboxylase (GAD), the rate-liminting enzyme in GABA biosynthesis, also plays an important role in maintaining GABA levels in the brain [31]. Hence, alteration of expression levels of this enzyme may change GABA transmission in the brain. We sought to determine GAD protein and GABA_A_ receptor subunit expression levels at the effective dosage of EFC and pentobarbital to determine the possible site of action by which EFC exerts its sleep-potentiating effects. GAD has two molecular forms (GAD_65_ and GAD_67_ with molecular weights of 65 kDa and 67 kDa, respectively), and we investigated expression levels of GAD_65_. GAD_65_ is responsible for vesicular GABA production; therefore this isoform is directly involved in GABA transmission at the synapse [32]. We also investigated the expression levels of GABA_A_ receptor *α*1-, *β*2-, and *γ*2-subunits. The most abundant GABA_A_ receptor subunit composition, *α*1*β*2*γ*2, is present in most brain regions, including the hippocampus, and these subunits are related to the hypnotic/sedative effect of GABA_A_ receptor ligands [30]. Our results showed that neither EFC nor pentobarbital treatment influenced expression of GABA_A_ receptor *β*2- and *γ*2-subunits; however pentobarbital decreased abundance of *α*1-subunits, and EFC increased levels of GAD. 

**Figure 5:**
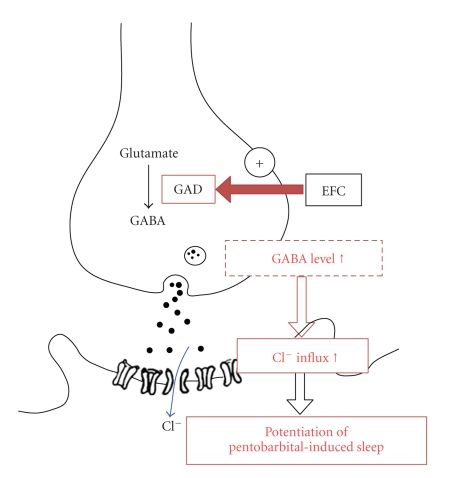
A proposed mechanism of EFC in augmenting pentobarbital-induced sleep. EFC administration will increase expression levels of GAD, the rate-limiting enzyme in GABA biosynthesis, thereby increasing GABA levels. This will promote Cl^−^ channel opening, causing hyperpolarization of the post-synaptic neuron. Hyperpolarization may result in potentiation of pentobarbital-induced sleep.

Many herbal preparations and a diversity of drugs used to promote sleep are known to act on GABA_A_ receptors [33]. Drugs acting on GABA_A_ receptors mainly act to increase synaptic inhibition either by directly activating GABA_A_ receptors or, more usually, by enhancing the action of other ligands on GABA_A_ receptors. Our results suggest that EFC has sleep-potentiating effects, which may be mediated by Cl^−^ channel opening (Figure 5). 

The search for novel plant-derived pharmacotherapies for psychiatric illness has progressed significantly in the past decade. A considerable number of herbal constituents whose behavioral effects and pharmacological actions have been well characterized may be good candidates for further investigations that may ultimately lead to clinical use of these constituents. The potential benefits of herbal remedies such as St. John's wort and Kava-kava in psychiatric practice have been addressed previously [34]. EFC may be another good candidate for the treatment of psychiatric illnesses, such as sleep disorders.

## 5. Conclusions

EFC enhanced hypnotic effects in pentobarbital-treated mice. This enhancement may result from Cl^−^ channel activation. Further investigation is needed to determine the effects of other EFC derivatives with strong pharmacological action.

## Figures and Tables

**Table 1:** Effects of EFC on sleep onset of mice treated by subhypnotic doses of pentobarbital (28 mg/kg, i.p.).

Group	Dose (mg/kg)	No. falling asleep/total	Sleep time (min)
Control	0	6/15	23.3 ± 1.8
Muscimol	0.2	13/14**	41.2 ± 4.2***
EFC	20	7/14	28.0 ± 4.0
	50	7/14	29.6 ± 3.2
	100	11/13*	35.9 ± 3.6*

Each value represents the mean (±S.E.M.) of 13–15 observations. *P < .05, **P < .01, ***P < .005 versus control.

## References

[1] Lai JP, Lim YH, Su J, Shen HM, Ong CN (2007). Identification and characterization of major flavonoids and caffeoylquinic acids in three Compositae plants by LC/DAD-APCI/MS. *Journal of Chromatography B*.

[2] Chu Q, Fu L, Guan Y, Ye J (2004). Determination and differentiation of Flos Chrysanthemum based on characteristic electrochemical profiles by capillary electrophoresis with electrochemical detection. *Journal of Agricultural and Food Chemistry*.

[3] Kim H, Lee YS (2005). Identification of new dicaffeoylquinic acids from *Chrysanthemum morifolium* and their antioxidant activities. *Planta Medica*.

[4] Jiang H, Xia Q, Xu W, Zheng M (2004). *Chrysanthemum morifolium* attenuated the reduction of contraction of isolated rat heart and cardiomyocytes induced by ischemia/reperfusion. *Pharmazie*.

[5] Miyazawa M, Hisama M (2003). Antimutagenic activity of flavonoids from *Chrysanthemum morifolium*. *Bioscience, Biotechnology and Biochemistry*.

[6] Ukiya M, Akihisa T, Yasukawa K (2001). Constituents of Compositae plants. 2. Triterpene diols, triols, and their 3-O-fatty acid esters from edible Chrysanthemum flower extract and their anti-inflammatory effects. *Journal of Agricultural and Food Chemistry*.

[7] Lin LZ, Harnly JM (2010). Identification of the phenolic components of chrysanthemum flower (*Chrysanthemum morifolium* Ramat). *Food Chemistry*.

[8] Beninger CW, Abou-Zaid MM, Kistner ALE (2004). A flavanone and two phenolic acids from *Chrysanthemum morifolium* with phytotoxic and insect growth regulating activity. *Journal of Chemical Ecology*.

[9] Hertog MGL, Feskens EJM, Hollman PCH, Katan MB, Kromhout D (1993). Dietary antioxidant flavonoids and risk of coronary heart disease: the Zutphen Elderly Study. *The Lancet*.

[10] Ohayon MM (2002). Methodology of a study on insomnia in the general population. *Encephale*.

[11] Morin CM, Gramling SE (1989). Sleep patterns and aging: comparison of older adults with and without insomnia complaints. *Psychology and aging*.

[12] Chistina Grobin A, Inglefield JR, Schwartz-Bloom RD, Devaud LL, Morrow AL (1999). Fluorescence imaging of GABA_A_ receptor-mediated intracellular [Cl^−^] in P19-N cells reveals unique pharmacological properties. *Brain Research*.

[13] Möhler H, Fritschy JM, Rudolph U (2002). A new benzodiazepine pharmacology. *Journal of Pharmacology and Experimental Therapeutics*.

[14] Tobler I, Kopp C, Deboer T, Rudolph U (2001). Diazepam-induced changes in sleep: role of the *α*1 GABA(A) receptor subtype. *Proceedings of the National Academy of Sciences of the United States of America*.

[15] Rudolph U, Crestani F, Möhler H (2001). GABA(A) receptor subtypes: dissecting their pharmacological functions. *Trends in Pharmacological Sciences*.

[16] Zhu S, Baker RC (1996). Effects of inhalation anesthetics on kainate-induced glutamate release from cerebellar granule cells. *Life Sciences*.

[17] Follesa P, Porcu P, Sogliano C (2002). Changes in GABA_A_ receptor *γ* subunit gene expression induced by long-term administration of oral contraceptives in rats. *Neuropharmacology*.

[18] Darias V, Abdala S, Martin-Herrera D, Luisa Tello M, Vega S (1998). CNS effects of a series of 1,2,4-triazolyl heterocarboxylic derivatives. *Pharmazie*.

[19] Wolfman C, Viola H, Marder M (1996). Anxioselective properties of 6,3’-dinitroflavone, a high-affinity benzodiazepine receptor ligand. *European Journal of Pharmacology*.

[20] West MR, Molloy CR (1996). A microplate assay measuring chloride ion channel activity. *Analytical Biochemistry*.

[21] Glowinski J, Iversen LL (1966). Regional studies of catecholamines in the rat brain. I. The disposition of [3H]norepinephrine, [3H]dopamine and [3H]dopa in various regions of the brain. *Journal of Neurochemistry*.

[22] Segal DS, Kuczenski R (1974). Tyrosine hydroxylase activity: regional and subcellular distribution in brain. *Brain Research*.

[23] Han H, Ma Y, Eun JS (2009). Anxiolytic-like effects of sanjoinine A isolated from *Zizyphi spinosi* Semen: possible involvement of GABAergic transmission. *Pharmacology Biochemistry and Behavior*.

[24] De Sousa FCF, Pereira BA, Lima VTM (2005). Central nervous system activity of yangambin from Ocotea duckei Vattimo (Lauraceae) in mice. *Phytotherapy Research*.

[25] Ma Y, Han H, Eun JS, Kim HC, Hong JT, Oh KIW (2007). Sanjoinine A isolated from *Zizyphi Spinosi* Semen augments pentobarbital-induced sleeping behaviors through the modification of GABA-ergic systems. *Biological and Pharmaceutical Bulletin*.

[26] Martínez AL, Domínguez F, Orozco S (2006). Neuropharmacological effects of an ethanol extract of the *Magnolia dealbata* Zucc. leaves in mice. *Journal of Ethnopharmacology*.

[27] Ma Y, Ma H, Jo YJ (2008). Honokiol potentiates pentobarbital-induced sleeping behaviors through GABA_A_ receptor Cl channel activation. *Biomolecules and Therapeutics*.

[28] Han H, Ma Y, Eun JS, Hong JT, Oh KIW (2008). Anxiolytic-like effects of cyclopeptide fraction alkaloids of *Zizyphi spinosi* semen: possible involvement of GABA_A_ receptors. *Biomolecules and Therapeutics*.

[29] Macdonald RL, Olsen RW (1994). GABA_A_ receptor channels. *Annual Review of Neuroscience*.

[30] Rudolph U, Möhler H (2006). GABA-based therapeutic approaches: GABA_A_ receptor subtype functions. *Current Opinion in Pharmacology*.

[31] Tillakaratne NJK, Medina-Kauwe L, Gibson KM (1995). Gamma-aminobutyric acid (GABA) metabolism in mammalian neural and nonneural tissues. *Comparative Biochemistry and Physiology A: Physiology*.

[32] Buddhala C, Hsu CC, Wu JY (2009). A novel mechanism for GABA synthesis and packaging into synaptic vesicles. *Neurochemistry International*.

[33] Chebib M, Johnston GAR (2000). GABA-activated ligand gated ion channels: medicinal chemistry and molecular biology. *Journal of Medicinal Chemistry*.

[34] Zhang ZJ (2004). Therapeutic effects of herbal extracts and constituents in animal models of psychiatric disorders. *Life Sciences*.

